# Development and Characterization of Solid Lipid Nanoparticles Loaded with a Highly Active Doxorubicin Derivative

**DOI:** 10.3390/nano8020110

**Published:** 2018-02-16

**Authors:** Barbara Stella, Elena Peira, Chiara Dianzani, Marina Gallarate, Luigi Battaglia, Casimiro Luca Gigliotti, Elena Boggio, Umberto Dianzani, Franco Dosio

**Affiliations:** 1 Dipartimento di Scienza e Tecnologia del Farmaco, University of Turin, Via P. Giuria 9, 10125 Turin, Italy; barbara.stella@unito.it (B.S.); elena.peira@unito.it (E.P.); chiara.dianzani@unito.it (C.D.); marina.gallarate@unito.it (M.G.); luigi.battaglia@unito.it (L.B.); 2 Dipartimento di Scienze della Salute, University of Eastern Piedmont Amedeo Avogadro, Via P. Solaroli 15, 28100 Novara, Italy; luca.gigliotti@med.unipo.it (C.L.G.); elena.boggio@med.unipmn.it (E.B.); umberto.dianzani@med.unipmn.it (U.D.)

**Keywords:** solid lipid nanoparticles, doxorubicin, anticancer agent, nanoassemblies, anticancer prodrugs

## Abstract

Solid lipid nanoparticles (SLNs) comprise a versatile drug delivery system that has been developed for the treatment of a variety of diseases. The present study will investigate the feasibility of entrapping an active doxorubicin prodrug (a squalenoyl-derivative) in SLNs. The doxorubicin derivative-loaded SLNs are spherically shaped, have a mean diameter of 300–400 nm and show 85% *w*/*w* drug entrapment efficiency. The effects on cell growth of loaded SLNs, free doxorubicin and the prodrug have been examined using cytotoxicity and colony-forming assays in both human ovarian cancer line A2780 wild-type and doxorubicin-resistant cells. Further assessments as to the treatment’s ability to induce cell death by apoptosis have been carried out by analyzing annexin-V staining and the activation of caspase-3. The in vitro data demonstrate that the delivery of the squalenoyl-doxorubicin derivative by SLNs increases its cytotoxic activity, as well as its apoptosis effect. This effect was particularly evident in doxorubicin-resistant cells.

## 1. Introduction

Anthracycline antitumor antibiotics are amongst the most powerful agents for the treatment of solid malignancies and can boast a wide range of antitumor activity. Doxorubicin hydrochloride (adriamycin) is the most commonly used and is effective in the treatment of carcinomas of the breast, lung, thyroid, ovary and soft tissue sarcomas [1]. However, anthracycline therapy is associated with significant general organ toxicity and especially myelosuppression, mucositis and cardiac toxicity [2].

Despite this widespread use of anthracyclines in cancer therapy, their limitations have continued to yield significant amounts of research. The main fields of anthracycline research are the continuing synthesis of novel anthracycline analogues to reverse drug resistance and reduce cardiotoxicity, while improving the delivery of anthracycline drugs is also an active field [3]. An important milestone in the field is found in the liposomal formulations Doxil^®^ (Caelyx^®^) [4] and Myocet^®^ [5]. Indeed, doxorubicin-loaded liposomes exhibit efficiencies comparable to those of the conventional anthracycline cytostatic agents, but with less cardiotoxicity. Nevertheless, the administration of long-circulating liposomes in clinical practice has been associated with palmar-plantar erythrodysesthesia (“hand-foot” syndrome), which may evolve into ulceration and epidermal necrosis if the chemotherapy cycle is not delayed [6].

Producing prodrug derivatives of doxorubicin is another way to approach improving activity against multidrug resistance and/or increasing cardiac tolerability [7]. An interesting doxorubicin prodrug, composed of a long lipophilic acyclic isoprenoid chain derived from squalene, has recently been created and tested. The conformation of squalene is greatly influenced by solvent polarity, so that in polar solvents, such as water, squalene takes a tightly-coiled conformation. This squalenoyl derivative of doxorubicin (SQ-Dox) is able to self-organize in water into unique “loop-train” elongated nanostructures with high stability [8].

The in vivo fate of squalenoyl nanoassemblies (NAs) has recently been described by Couvreur and co-workers, who have highlighted the prominent role played by endogenous lipoproteins (LDL) in the delivery of squalenoyl NAs in the bloodstream; LDL adsorb onto the NAs, which then disassemble, releasing squalenoyl bioconjugates that insert into lipoproteins. Thus, the pharmacokinetics and biodistribution are dependent on the LDL route [9,10].

Solid lipid nanoparticles (SLNs) are dispersed systems that display sizes ranging from 1–1000 nm and represent an alternative to polymeric particulate carriers. They are composed of physiological or biocompatible lipids or lipid molecules with a history of safe use in therapy, and they are generally suitable for intravenous administration [11]. Some authors have found that SLNs, which were loaded with doxorubicin, were able to overcome P-glycoprotein (Pgp)-mediated multidrug resistance (MDR), both in vitro in resistant leukemia cells and in vivo in the murine leukemia mouse model [12,13]. The results suggested that SLNs, which have attracted huge research interest from the field of site-specific drug targeting, including brain delivery [14], over the last few decades may be able to provide the potential to deliver anticancer drugs for the treatment of Pgp-mediated MDR. Indeed, SLNs have been proposed for use as innovative chemotherapeutic agent vehicles for experimental glioblastoma multiforme (GBM) treatment, owing to their ability to enhance drug uptake by cells and evade the Pgp efflux system. It is well known that SLNs are subjected to endocytosis by endothelial cells and that they can also be exploited for passive and, if opportunely surface-modified, active targeting to the brain [15]. Indeed, in order to increase cancer cell-selective cytotoxicity, one strategy is to surface-engineer drug delivery systems for molecular targeting. This strategy, which exploits the differences between cancer cells and healthy cells, has recently been adopted for SLN anticancer drug delivery. A folate receptor-targeted lipid nanoparticle system has been developed for the delivery of a paclitaxel prodrug [16]. Targeted nanoparticles showed elevated antitumor activity in mice bearing the M109 tumor with the marker folate receptor. Functionalization with a chimera peptide (Apo E), having a lipophilic moiety and an aminoacidic sequence for very low-density lipoprotein (VLDL) receptor binding (ApoE), was exploited in order to target SLNs to the VLDL receptor. Enhancement of brain accumulation of lipophilic methotrexate prodrug by ApoE-conjugated SLNs was evident especially at longer post-administration times, when compared with non-functionalized SLNs [17]. Moreover, as described in the literature, loading drugs into SLNs can often lead to their physico-chemical and hydrolytic stability being improved, as previously seen in fatty acid SLN-entrapped curcumin [18].

Thus, we herein investigate the feasibility of encapsulating the active lipophilic derivative SQ-Dox into SLNs in an effort to evaluate the potential of SLNs in releasing the active drug into tumor cells and with a view toward conferring improved properties to this anticancer prodrug. The lipid moiety in the conjugate can significantly enhance drug loading into the hydrophobic components of delivery carriers and thus generates formulations with improved drug loading and superior stability [19].

The SLNs proposed in this study are prepared using the technique of fatty acid coacervation and are stabilized by biodegradable polymers with hydrophilic characteristics, which, once adsorbed onto the SLN surface, can prolong in vivo half-life by preventing opsonization.

For the above-mentioned purpose, SLNs were prepared and extensively characterized with regards to their physico-chemical properties, their loading capacity and their cytotoxic potency.

## 2. Materials and Methods

### 2.1. Materials

Doxorubicin and daunorubicin hydrochloride were purchased from APAC Pharmaceutical LLC (Columbia, MD, USA). Behenic acid sodium salt (Na-BA) was purchased from Nu-Chek Prep, Inc. (Elysian, MN, USA). Squalene, polyvinyl alcohol (PVA 9000), acetonitrile, dimethylformamide, dioxane, dimethylsulfoxide, lauric acid, tetrahydrofuran, trifluoroacetic acid and dichloromethane were obtained from Sigma (Dorset, UK). Hydrochloric acid, sodium hydroxide and monobasic sodium phosphate were obtained from Merck (Darmstadt, Germany). Methanol, thionyl chloride, diisopropyl ether, diethyl ether and ethanol were obtained from Carlo Erba (Val De Reuil, France). TLC plates and silica gels for chromatography were purchased from Merck, and ultrapure (Type 1) water was obtained using a MilliQ system (Millipore, Bedford, MO, USA). 2,3-bis[2-methoxy-4-nitro-5-sulphophenyl]-2H-tetrazolium-5-carboxanilide (MTT) and crystal violet were purchased from Sigma (Dorset, UK).

### 2.2. Methods

#### 2.2.1. Synthesis of SQ-Dox and Preparation of SQ-Dox NAs

The synthesis of doxorubicin-14-squalenate [8] proceeded as follows: Trimethyl orthoformate (0.20 mL, 1.83 mmol) was added to a solution of daunorubicin hydrochloride (0.20 g, 0.35 mmol), dissolved in methanol/1,4-dioxane (1:2 (*v*/*v*), 12 mL). The reaction mixture was then stirred at room temperature for 20 min. A Br_2_/CHCl_3_ (1:9 (*w*/*v*), 0.68 mL, 0.43 mmol) solution was then added to this mixture. After stirring for 40 min at 30 °C, the resulting solution was poured into dry ether (200 mL), and the solid residue was filtered and washed with ether (50 mL × 3). The solid was re-crystallized from acetone/ether (1:1 (*v*/*v*), 10 mL), filtered off, washed with ether and dried over P_2_O_5_ to give 14-bromo-daunorubicin (0.19 g, 84%), as a red solid with a melting point of 176–177 °C. The 14-bromo-daunorubicin (415.6 mg, 0.625 mmol) and 1,1′,2-tris-norsqualenoic acid (500 mg, 1.25 mmol) were dissolved in acetone (50 mL) under inert argon atmosphere. Potassium carbonate (260 mg, 1.875 mmol) was then added, and the reaction mixture was stirred at room temperature for 48 h (dark). The solvent was evaporated and the crude product purified using silica gel flash column chromatography (95:5, CH_2_Cl_2_–MeOH), to give a red powder (249.5 mg, 43%). The target compound, dissolved in anhydrous THF (240 mg in 2 mL), was then converted to its hydrochloride salt via the addition of an anhydrous, titrated 4 M solution of HCl in dioxane (1.2 eq., 0.075 mL) and subjecting it to stirring at −20 °C for 2 h. The solvents were then removed, and the red solid product was further purified by washing with diisopropyl ether. The yield of doxorubicin-14 squalenate hydrochloride (SQ-Dox) was 171.9 mg (70%). The purity of SQ-Dox was checked by silica TLC-eluted CH_2_Cl_2_: MeOH:HCOOH:H_2_O (88:15:2:1, Rf 0.5) and by HPLC-MS. ^1^H NMR (methanol-d4): 8.02 (d, 1H, H-3), 7.87 (d, 1H, H-1), 7.70 (t, 1H, H-2), 5.46 (s, 1H, H-10), 5.3–5.25 (m, 2H, H-14a, H-14b) and 5.20 [s, 5H, C(SQ-H)], 5.19 (s, 1H, H-7), 4.15 (q, 1H, H-50), 4.01 (s, 3H, OCH_3_), 3.74 (m, 2H, H-30, H-40), 3.24 (d, 1H, H-10), 3.00 (d, 1H, H-10), 2.43 (m, 1H, H-8), 2.29 and 2.35 (s, 4H, CH_2_ SQ), 2.13 (m, 1H, H-8), 2.03 (m, 16H, CH_2_ SQ), 1.97 (m, 1H, H-20), 1.82 (m, 1H, H-20), 1.71 (m, 18H, C(SQ)-CH_3_), 1.29 (d, 3H, CH_3_); HPLC: the Waters XTerra RP-18 column was eluted with water, methanol (starting at 50:50 for 7 min, after which the gradient was moved to 100% methanol, 15 min), plus formic acid 0.05%. Elution time was 28.95 min. The elution was monitored at 234 and 480 nm using a Waters 2996 Photodiode Array detector. ESI MS (Waters micromass), *m*/*z* calculated for [C_54_H_71_NO_12_ + H]^+^: 927.14. Found 927.2 (MH^+^). Elemental analysis calculated: C 67.38%, H 7.54%, Cl 3.68%, N 1.46%. Found: C 67.42%, H 7.61%, Cl 3.67%, N 1.42%.

SQ-Dox NAs were prepared using the nanoprecipitation method, and the freshly prepared formulation was used for further in vitro studies. Briefly, 500 μL of the tetrahydrofuran solution of SQ-Dox (4 mg/mL) were added dropwise under stirring (500 rpm) to 1 mL of a 1 mM phosphate saline buffer of pH 7.4. The precipitation of the SQ-Dox NAs occurred spontaneously. The organic solvent was completely evaporated under vacuum to obtain an aqueous suspension of pure SQ-Dox NAs (final concentration 2 mg/mL). SQ-Dox NAs were stored at 4 °C.

#### 2.2.2. Preparation SQ-Dox-Loaded SLNs

Appropriate amounts of Na-BA (1% *w*/*w*) and PVA 9000 (2% and 3% *w*/*w* in the SLN1 and SLN2 preparations, respectively), used as polymeric steric stabilizers [20], were dispersed in 5 mL deionized water, and the mixture was then heated under stirring (300 rpm), at just above the Krafft point of Na-BA (75 °C), to obtain a clear micellar solution. A selected acidifying solution (100 µL 1 M NaH_2_PO_4_ + 160 µL 1 M HCl) was then added dropwise until pH 3.5–4.0 was reached. The obtained suspension was then cooled in a water bath under stirring at 300 rpm until the SLNs completely precipitated. Drug-loaded SLNs were prepared as described for blank SLNs (empty SLNs), plus the addition of 200 µL of a SQ-Dox solution (10 mg/mL in methanol:2-propanol 2:1 *v*/*v*), directly into the 2.5-mL SLN suspension, which was heated to just above the melting point of BA (80 °C). The sample was then cooled in a water bath under stirring at 300 rpm until a temperature of 15 °C was reached. In order to remove traces of any non-entrapped drug, SLN preparations were purified by gel centrifugation on Bio-gel P-6DG (pore size 90–180 µm, nominal exclusion limit 6000 Da). All procedures were performed under a horizontal laminar flow hood, and SLN preparations were stored at 4 °C.

#### 2.2.3. NA and SLN Characterization

##### 2.2.3.1. Size and Zeta Potential Measurements

The average diameter and polydispersity index (PDI) of the NAs and SLNs were determined using quasi-elastic light scattering (QELS) and a nanosizer (Brookhaven, NY, USA), at a fixed angle of 90° and a temperature of 25 °C. The various dispersions (NAs or SLNs) were 1:20 diluted with MilliQ water before analysis. SLNs and NAs were also diluted with the various growth media described later in the cytotoxicity studies. Each value reported is the average of five measurements.

In order to determine electrophoretic mobility, NA and SLN samples were opportunely diluted with 0.1 mM KCl and placed in the electrophoretic cell of the nanosizer, where an electric field of 15.24 V/cm was established. Each sample was analyzed in triplicate. Zeta potential values were calculated using the Smoluchowski equation.

Particle size, polydispersity index and zeta potential were determined 1 h after suspension preparation. For size stability studies vs. time, samples were stored at 4 °C. All data were determined in triplicate. 

Optical microscopy, equipped with a fluorescent lamp (Leica DM 2500, Solms, Germany) at 630× magnification, was used to determine whether the SQ-Dox derivative was effectively localized within the SLN dispersion.

Entrapment efficiency (EE%) determination was performed as follows: a 1-mL SLN suspension was centrifuged for 15 min at 62,000× *g*, and the precipitate was then washed twice with 1 mL ethanol:water (30:70 *v*/*v*), to eliminate the adsorbed drug. The solid residue was dissolved in dimethylsulfoxide:dichloromethane (1:1 *v*/*v*). Then, 0.1 mL water were added in order to precipitate the lipid matrix, and the supernatant was injected into a HPLC for SQ-Dox derivative determination, as described later. The SQ-Dox EE% was calculated as the ratio between the amount of drug in the SLNs and that in the starting micellar solution × 100.

Loading capacity (DL%) is the amount of drug loaded per unit weight of SLNs, indicated as the percentage of mass.

##### 2.2.3.2. Differential Scanning Calorimetry 

DSC studies were performed using a Perkin Elmer DSC-7 (Perkin Elmer, Walthman, MS, USA) and Pyris Version 3.7.1 software (Perkin Elmer, Walthman, MS, USA). The DSC measurements were carried out on the following samples: unloaded SLNs, SQ-Dox loaded SLNs and free SQ-Dox. A weighed amount of each lyophilized sample was transferred to a calorimetric pan, hermetically sealed and submitted to DSC analysis. Heating curves were recorded by transferring an amount of the various samples to aluminum pans with a scan rate of 10 °C/min at a temperature range of 40 °C to 250 °C using an empty aluminum pan as the reference.

##### 2.2.3.3. Atomic Force Microscopy 

AFM observation was carried out using a Park XE 100 atomic force microscope (Park Instruments, Sunnyvale, CA, USA). AFM images were obtained via measurement of the interaction forces between the tip (Mikromash NSC15) and the sample surface. The experiments were conducted at room temperature (20 °C), at atmospheric pressure (760 mmHg), and were operated in non-contact mode, in which the space between the tip and the sample was between 10 and 100 Å, and the total force was very low. The resonant frequencies of this cantilever were found to be around 250 kHz. The samples were diluted in water (1:10 *v*/*v*), immediately before analyses, to provide a less sticky fluid to analyze. SLN droplets of constant volume (20 µL) were deposited onto a small mica disk with a diameter of 1 cm. The water excess was removed using a paper filter after 2 min.

##### 2.2.3.4. SQ-Dox Stability Evaluation: HPLC Analysis

In order to define the stability of SQ-Dox after SLN preparation and storage, HPLC analyses were performed using the YL9100 HPLC system equipped with a YL9110 quaternary pump, a YL 9101 vacuum degasser and a YL 9160 PDA detector linked to YL-Clarity software Version 3.0.4.444 for data analysis (Young Lin, Hogye-dong, 258 Anyang, Korea). A LiChrospher 100 RP8 5 μm 80 × 4.6 mm column (Merck) was eluted with acetonitrile (A) and phosphate buffer (50 mM at pH = 2 adjusted by HCl (0.01 N) (B) according to the following gradient system (30% A–70% B to 90% A–10% B in 15 min at a flow rate of 1 mL/min; total run of 25 min). The UV-Vis detector was set at λ 480 nm. Rt was 16 min.

#### 2.2.4. In Vitro Biological Evaluations

##### 2.2.4.1. Cell Cultures

Cells and culture conditions: A2780 (human ovarian carcinoma) and A2780 Dox-resistant (A2780 res) cell lines were obtained from ATCC (Milan, Italy). The tumor cell lines were grown as monolayers in culture dishes in RPMI 1640 medium (Invitrogen, Burlington, ON, Canada), supplemented with 10% penicillin-streptomycin (Invitrogen) and 10% fetal calf serum (Euroclone, Milan, Italy), in a humidified atmosphere at 37 °C and 5% CO_2_. To maintain resistance, A2780 Dox-resistant cell medium was supplemented with 1 μg/mL of Dox once a week.

##### 2.2.4.2. Cell Viability Assay

Cells (3 × 10^3^/well) were seeded in 96-well plates and incubated at 37 °C, in 5% CO_2_, for 24 h. Cells were then either treated with varying concentrations of Dox, SQ-Dox, SQ-Dox-loaded SLNs (10^−8^–10^−5^ M as Dox) or empty SLNs. After 72 h of incubation, the number of viable cells was evaluated using the 2,3-bis[2-methoxy-4-nitro-5-sulphophenyl]-2H-tetrazolium-5-carboxanilide (MTT) inner salt reagent at 570 nm, as described in the manufacturer’s protocol. The controls (i.e., cells that had received no drug) were normalized to 100%, and the readings from the treated cells were expressed as % of viability inhibition. Six replicates were used to determine each data point, and five different experiments were performed.

##### 2.2.4.3. Colony-Forming Assay

A2780 and A2780 res cells (3 × 10^3^/well) were seeded into six-well plates and treated with either Dox, SQ-Dox, SQ-Dox-loaded SLNs (10^−9^–10^−5^ M as Dox) or empty SLNs. The medium was changed after 72 h, and cells were cultured for an additional 10 days. Cells were subsequently fixed and stained with a solution of 80% crystal violet and 20% methanol. Colonies were then photographed and counted using Gel Doc equipment (Bio-Rad Laboratories, Milan, Italy). The cells were then perfectly washed, and 30% acetic acid was added to induce the complete dissolution of the crystal violet. Absorbance was recorded at 595 nm using a 96-well-plate ELISA reader. Five different experiments were performed.

##### 2.2.4.4. Cell Death Analysis

A2780 and A2780 res cell lines were either treated with a variety of concentrations of Dox, SQ-Dox, SQ-Dox-loaded SLNs, empty SLNs or left untreated. Cells were harvested 72 h after treatment, washed with PBS and subsequently resuspended in annexin-V binding buffer (BD Biosciences, San Jose, CA, USA), supplemented with 1:100 APC-conjugated annexin-V (BD Biosciences). Cells were analyzed using a FACS Calibur cytometer (BD, Bioscience). Subsequently, caspase-3 activity was assessed in cell lysates using a fluorometric assay (MBL, Watertown, MA, USA), according to the manufacturer’s instructions.

#### 2.2.5. Statistical Analysis

Data were expressed as the mean ± SEM (standard error mean = standard deviation/number of replicates). Statistical analyses were performed using GraphPad Prism 3.0 software (San Diego, CA, USA), as well as the one-way ANOVA and Dunnett tests. Values of *p* < 0.05 were considered statistically significant.

## 3. Results

### 3.1. NA and SLN Preparation and Characterization

The nanoformulations containing SQ-Dox were prepared as described above. SLNs were prepared using the coacervation method, based on the precipitation of fatty acids. Precipitation occurred when the pH of a fatty acid-alkaline salt micellar solution was lowered by acidification in the presence of an appropriate polymeric stabilizer, which was able to confer hydrophilicity to the SLN surface [21]. Behenic acid sodium salt was chosen as the component of the lipid matrix as it has not shown any evidence of detectable cytotoxicity [22]. SQ-Dox NAs were prepared by the nanoprecipitation process obtained by solvent displacement. All the formulations were prepared and checked in triplicate. The mean diameters (± standard error, S.E.), PDI and zeta potential of SLNs and SQ-Dox NAs were checked first using quasi-elastic light scattering (Table 1). SQ-Dox nanostructures displayed a low mean diameter, which was smaller than that of empty SLNs. The incorporation of the drug into SLNs increased the mean diameter of lipid nanoparticles to the 300–400-nm range. Even if the zeta potential values fall down into the neutrality range of charged systems, small differences probably due to surface modifications can be observed. The zeta potential of the empty SLNs prepared by coacervation is around 0 mV [18], because polyvinyl alcohol (PVA 9000), which was used as a stabilizer, locates itself externally, partially screening the surface charge [21]. In the SQ-Dox-loaded SLNs, the zeta potential value also depended on the drug arrangement in the lipid matrix [23], meaning that the slightly positive value shown by SQ-Dox SLNs can be due to the partial arrangement of the drug on the outside of the SLN surface. The slight difference between zeta potential of SLN1 and SLN2 can be due to the different amount of PVA (higher in SLN2) partially shielding the SLN surface. Anyway, these small differences probably do not contribute to significant variation of SLN stability.

The stability of the suspensions was checked by measuring mean size, both after long storage times (up to 45 days), at 4 °C in water and in cell medium (RPMI 1640 supplemented with 10% penicillin-streptomycin (Invitrogen) and 10% fetal calf serum) for incubation times up to 72 h. Particle diameters were measured in water at a range of times, up to 45 days, and were practically unmodified. Growth media do not seem to influence SLN dimensions, as all formulations maintained their size (±5%), while the size of SQ-Dox NAs was significantly affected after 72 h at 37 °C in cell medium (Table 1). The stability of the active agent in each phase of the SLN preparation and storage was assessed by HPLC analysis. No significant amounts of SQ-Dox degradation products were observed (data not shown).

The amount of SQ-Dox in SLNs, as prepared by dispersion in 2% *w*/*w* (SQ-Dox SLN1) and 3% *w*/*w* (SQ-Dox SLN2) PVA 9000 aqueous solution, is expressed as EE%. This value was calculated after SLN centrifugation and washing with ethanol:water 30:70 *v*/*v*. Encapsulation efficiency was significantly influenced by PVA 9000 content; when the stabilizer percentage was increased from 2%–3% *w*/*w*, a decrease in drug entrapment from 85–50 ± 3% (*w*/*w*) was noted (Table 1). This was probably caused by PVA 9000 forming micelles in the aqueous phase, which were able to solubilize SQ-Dox. Nevertheless, SLNs showed good SQ-Dox loading capacity ranging from 4.0% for SQ-Dox SLN2 to 6.8% for SQ-Dox SLN1. Due to higher drug loading, the SLN1 formulation was chosen for further characterizations and tests on cancer cell lines.

The behavior of SQ-Dox was evaluated at the same temperature and pH conditions as the SLN preparation, since this derivative is able to self-assemble in water to form NAs. The obtained particles showed micrometric dimensions under QELS measurement, and large aggregates were observed under the microscope, meaning that the concomitant self-assembly of the conjugate can be ruled out.

**Figure 1:**
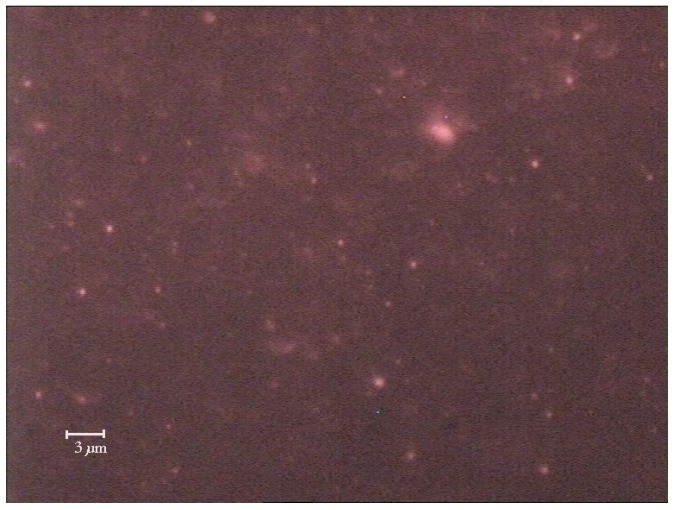
Optical microscopy images of SQ-Dox-loaded SLNs, Formulation 1.

SLN microphotographs are reported in Figure 1. Only particles with a mean diameter higher than 500 nm were clearly visualized, according to optical microscopy resolution power. Anyway, the presence of drug crystals was excluded by observation under polarized light. Moreover, when SQ-Dox was submitted to the same procedure used for SLN preparation in the absence of the lipid matrix, no self-assembled structures were evidenced. The observation of SLN suspensions under the optical microscope, although not a conclusive test for the physico-chemical characterization of SLNs, confirmed their formation. The possible presence of micellar structures was not detectable with optical microscopy, owing to the small size of micelles. The use of fluorescent light allowed SQ-Dox association with SLNs to be identified; dispersions of fluorescent SLNs were observed in both suspensions.

We move now to DSC measurements. A thermogram of SQ-Dox NAs reveals a broadened transition peak at a temperature (T_m_) of around 153 °C (Table 2). A strongly lowered transition peak was observed, with a shift at lower temperature (about 141 °C), when the conjugate was incorporated into SLNs. This behavior is probably due to conjugate incorporation into the lipid matrix and to interactions with the nanoparticle structure. Moreover, thermograms of SLNs reveal a sharp melting peak at 75 °C, which is a little lower than that of raw BA.

According to Siekmann and Westesen [24], melting point decreases in SLN colloidal systems can be caused by the colloidal dimensions of the particles and, in particular, their high surface-to-volume ratio. The melting point falls when bulk matrix material is turned into SLNs; the presence of impurities, surfactants and stabilizers could also have an influence on this phenomenon [25].

The BA-SLNs’ peak was also maintained in the presence of the conjugate, which indicates that the nanoparticle structure was unmodified (data not shown).

**Figure 2:**
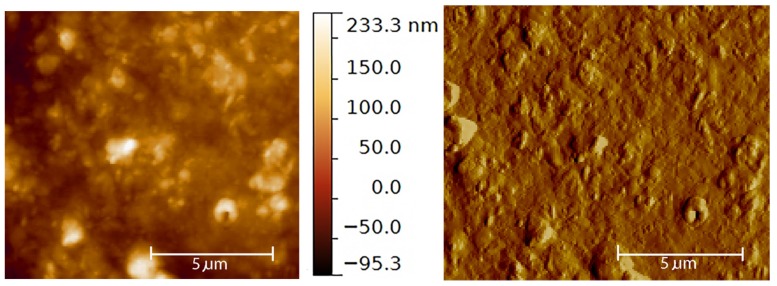
Underwater AFM images, performed in non-contact mode, of the SQ-Dox SLN1 formulation after sample deposition on a mica support: topography on the left and amplitude on the right.

The morphology of SQ-Dox SLN1 was investigated using AFM. A topography AFM image is presented in Figure 2. The structures evident in the image confirm the size measured by QELS, although the presence of a certain degree of aggregation can be observed. We also noted the surface roughness of the SLNs. Further investigation with cryo-transmission electron microscopy could be useful to clarify the morphology.

### 3.2. In Vitro Evaluations

#### 3.2.1. SQ-Dox NA and SQ-Dox SLN1 Inhibition in Cell Proliferation Tests

We compared the ability of Dox, SQ-Dox NAs, SQ-Dox SLN1 and empty SLNs to inhibit the growth of A2780 and A2780 res cells. Cells were cultured either in the presence or the absence of titrated amounts (10^−8^–10^−5^ M as Dox equivalent) of each sample for 72 h, and the number of viable cells was then assessed using the MTT assay.

**Figure 3:**
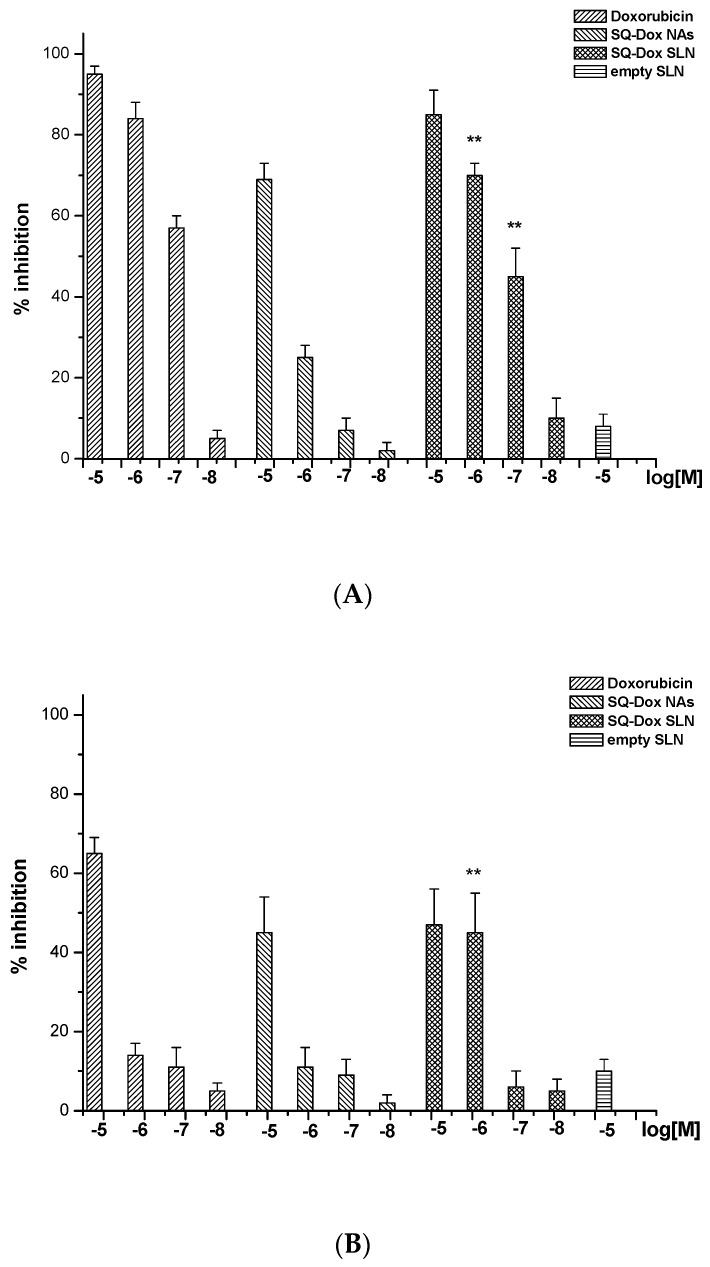
Inhibition of A2780 (**A**) and A2780 res (resistant) (**B**) cell proliferation following Dox, SQ-Dox NAs and SQ-Dox SLN1 treatment. Cells were treated with increasing concentrations (expressed as Dox equivalent) of compounds and empty SLNs for 72 h. Six replicates were used to determine each data point, and five different experiments were performed. One-way ANOVA and Dunnett’s test revealed statistically-significance differences (** *p* < 0.01) between the effects caused to A2780 res cells of the loaded SLNs vs. SQ-Dox treatments.

Figure 3 shows the inhibition of cell proliferation induced by Dox, SQ-Dox NAs and SQ-Dox SLN1. The effect was concentration dependent, with an inhibition of 70–90% observed at the highest concentrations. While Dox is more efficient than SQ-Dox, SQ-Dox SLN1 induces an inhibitory effect that is similar to that of Dox in the A2780 cell line (Panel A). A2780 res cell proliferation was naturally affected only by the higher concentrations of Dox and SQ-Dox, which induced an inhibition of around 50%, while SQ-Dox SLN1 also remained effective at a concentration of 10^−6^ M (Panel B). Cells treated with empty SLNs displayed the same effects as those experienced by untreated cells.

**Figure 4:**
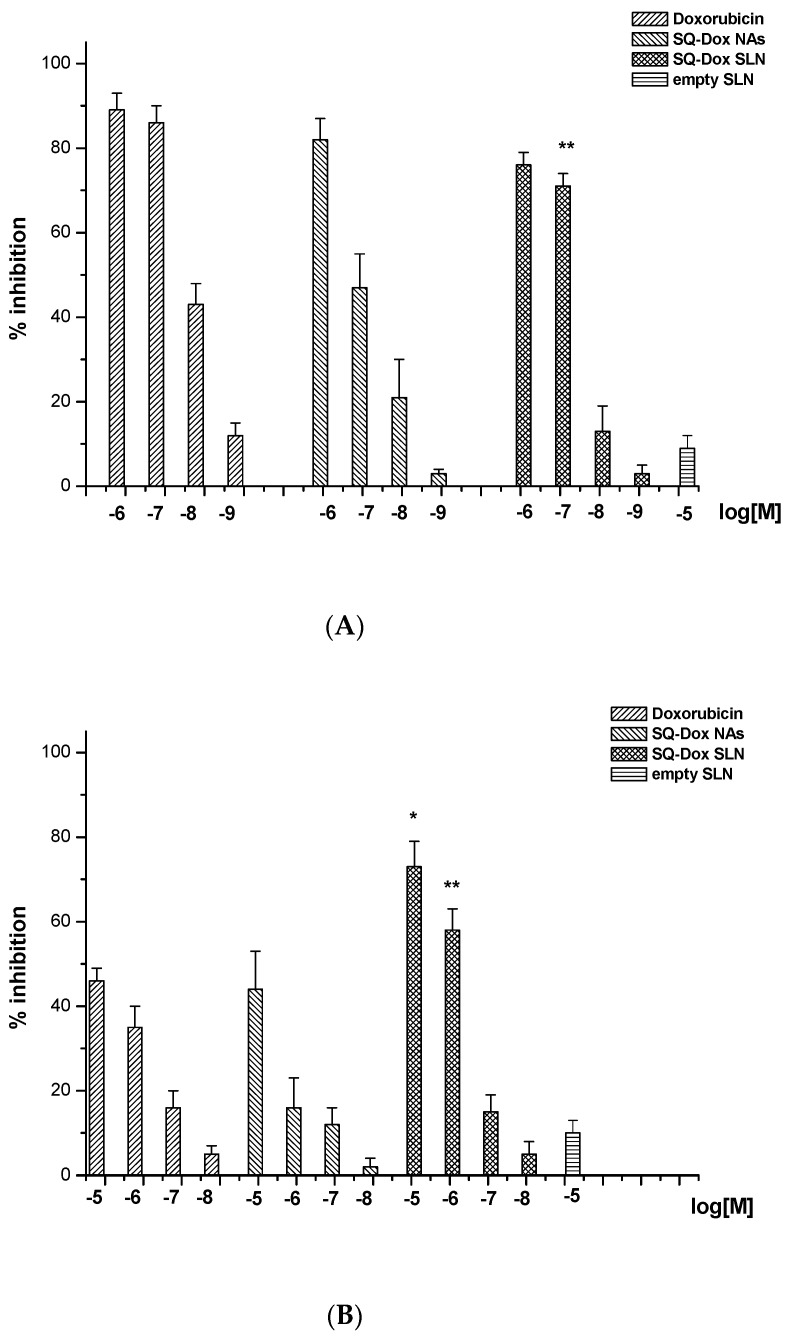
The effects of Dox, SQ-Dox NAs and SQ-Dox SLN1 treatment on cell clonogenicity were tested by a colony-forming assay of A2780 (**A**) and A2780 res (**B**) cell lines. One-way ANOVA and Dunnett’s tests were performed on five different experiments, revealing statistically-significance differences (* *p* < 0.05; ** *p* < 0.01) in SQ-Dox SLN1 vs. Dox-treated cells.

Clonogenic survival assays were performed to validate these findings (Figure 4). Cells were seeded onto six-well plates and treated with titrated amounts (10^−8^–10^–5^ M as Dox equivalent) of each sample. The culture medium was changed after 72 h, and cells were cultured for an additional 10 days in the absence of the compounds. The results showed that treatment with SQ-Dox SLN1 inhibited the A2780 cell line’s ability to form colonies, similarly to Dox. However, SQ-Dox was slightly less efficient at the lowest concentrations (Panel A). SQ-Dox SLN1 was even more effective in the A2780 res cell line, proving that Dox is slowly released from the SLNs, which protects the drug from degradation and prevents efflux pump extrusion (Panel B).

#### 3.2.2. SQ-Dox NAs and SQ-Dox SLN1 Increase Caspase-3 Activity In Vitro

**Figure 5:**
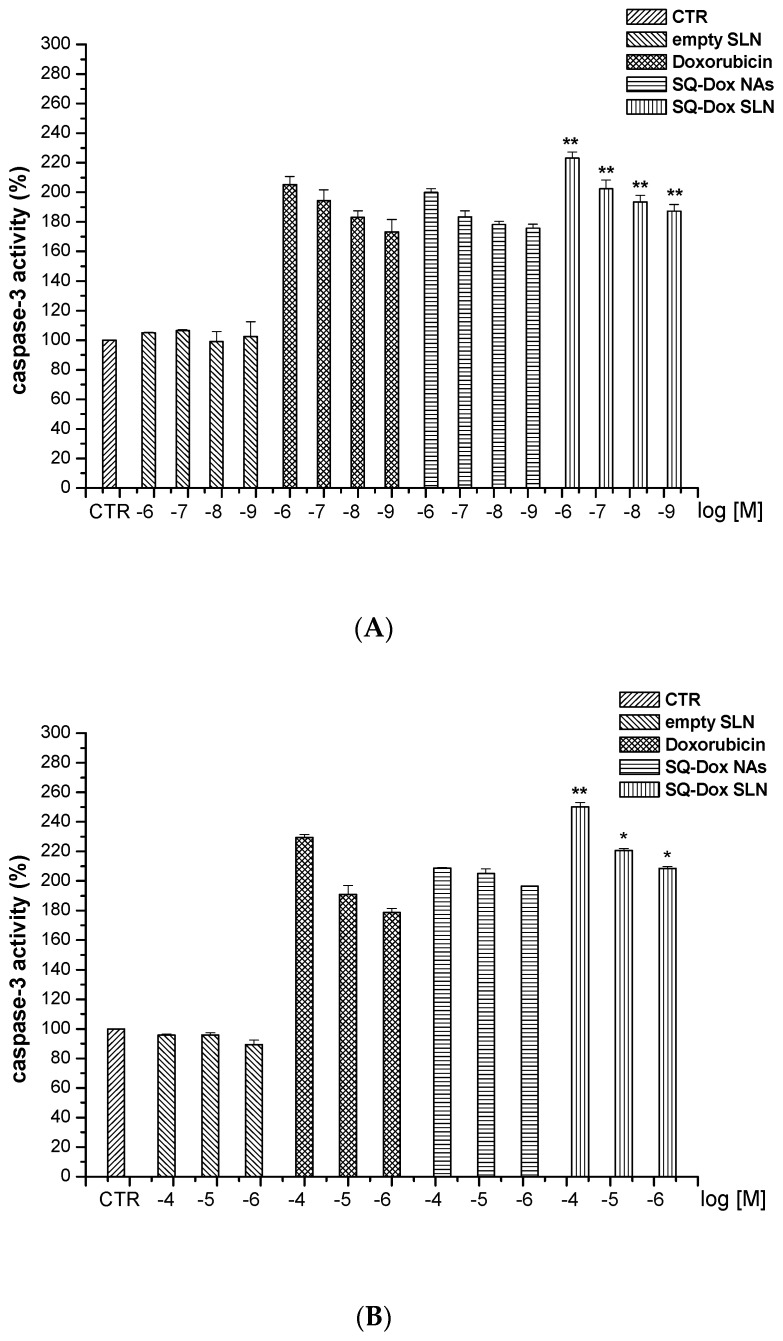
Levels of caspase-3 activity after Dox, SQ-Dox NAs and SQ-Dox SLN1 treatment. Caspase-3 activity was evaluated in A2780 (**A**) and A2780 res (**B**) cell lines cultured for 72 h in either the presence or absence of Dox, SQ-Dox NAs, SQ-Dox SLN1 or empty SLNs. Results are expressed as % of relative caspase activity, calculated as follows: (result displayed by each treatment/result displayed by untreated cells). * *p* < 0.05 and ** *p* < 0.01, vs. Dox-treated cells at the same concentrations, from three independent experiments.

The levels of cell death, evaluated by the activation of caspase-3, in A2780 and A2780 res cell lines were evaluated after treatment with Dox, SQ-Dox NAs, SQ-Dox SLN1 and empty SLNs at a variety of concentrations (Figure 5). Treatment with SQ-Dox NAs and SQ-Dox SLN1, at all concentrations tested, significantly increased caspase-3 activity in A2780 cells, as compared to Dox-treated cells. However, the SQ-Dox SLN1 formulation showed the highest efficacy, compared to SQ-Dox cells treated at the same concentrations, at all the concentrations tested. Notably, SQ-Dox SLN1 maintained its efficacy compared to SQ-Dox at 10^−9^ M (Panel A).

SQ-Dox and loaded SLNs significantly increased caspase-3 activity in the A2780 res cell line, compared to Dox-treated cells, at all the concentrations tested. However, the loaded SLN formulation exerted a higher efficacy, at a concentration of 10^−5^ M, than that exerted on SQ-Dox-treated cells at the same concentration (Panel B). Treatment with empty SLNs displayed the same effects as those observed in untreated cells.

#### 3.2.3. SQ-Dox NAs and SQ-Dox SLN1; Levels of Annexin-V

**Figure 6:**
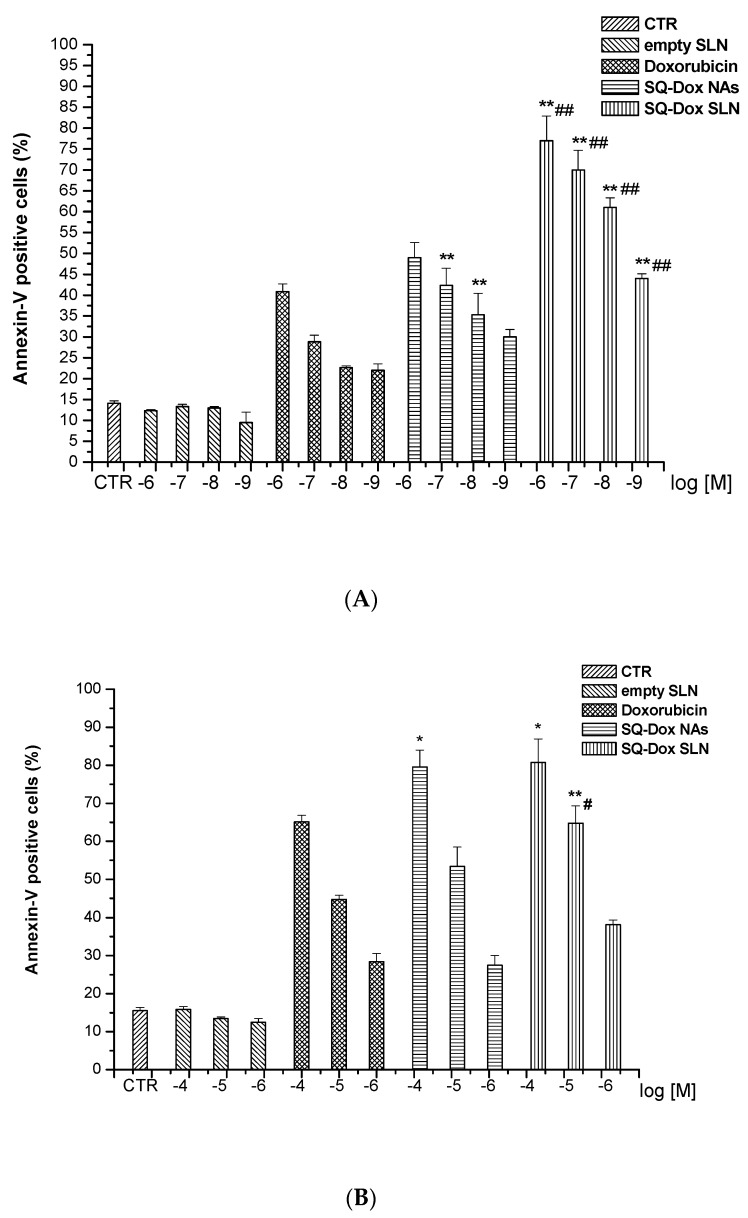
Levels of annexin-V-positive cells after Dox, SQ-Dox NAs, SQ-Dox SLN1 treatment. Annexin-V activity was evaluated in A2780 (**A**) and A2780 res (**B**) cell lines cultured for 72 h in either the presence or absence of Dox, SQ-Dox NAs, SQ-Dox SLN1 or empty SLNs. * *p* < 0.05 and ** *p* < 0.01, vs. Dox-treated cells at the same concentrations; # *p* < 0.05 and ## *p* < 0.01 vs. SQ-Dox-treated cells at the same concentrations, from three independent experiments.

A further assessment of SQ-Dox SLN1’s ability to induce cell death was obtained by analyzing annexin-V staining, for the detection of both apoptotic and necrotic cells, in A2780 and A2780 res cells cultured in the presence and absence of titrated amounts of Dox and SQ-Dox NAs, as well as loaded and empty SLNs for 72 h (Figure 6).

The results obtained show that SQ-Dox NAs and SQ-Dox SLN1 induced higher annexin-V staining in the A2780 cell line than free Dox (Panel A). Moreover, loaded SLNs were significantly more effective than SQ-Dox at increasing annexin levels at all tested concentrations, which is in line with the findings obtained from caspase-3 analyses.

SQ-Dox NAs and SQ-Dox SLN1 similarly induced higher annexin-V staining in the A2780 res cell line than free Dox at the highest concentrations. However, only 10^−5^ M SQ-Dox SLNs reported a significant increase in annexin-V levels, when compared to SQ-Dox NAs at the same concentrations (Panel B).

## 4. Discussion

SLNs are a category of versatile drug delivery systems that have been studied in the biomedical field for several years. One of the main reasons for the fast development of SLNs is their ability to effectively deliver both lipophilic and even smaller nanoparticles in such a way as to combine, as frequently reported in literature, therapeutic and diagnostic behavior. From an industrial point of view, other advantages can be found in the fact that their preparation often does not require organic solvents, that they can be easily sterilized and that the production of these lipid nanocarriers can be easily scaled up [26]. In particular, the preparation of SLNs using the fatty acid coacervation technique (FACT), which allows the incorporation of drugs in a micellar solution of FA alkaline salts, does not require complex equipment and can be easily moved for industrial application [27]. This method is based on the slow interaction between a micellar solution of a sodium salt of a fatty acid and an acid solution (coacervation solution), in the presence of a proper amphiphilic polymeric stabilizing agent; the sudden lowering of pH causes SLN precipitation. Among the various fatty acids available, behenic acid was chosen as the lipid matrix, because, contrary to stearic and palmitic acid, it does not cause any detectable cytotoxicity in a number cell lines [28]. This method has been widely used to entrap both lipophilic anticancer agents (directly) and hydrophilic drugs, such as Dox, after the formation of hydrophobic ion pairs [29]. To further improve stability, some Dox lipophilic prodrugs have recently been prepared and efficiently incorporated in SLNs [21].

The squalenoyl derivative used in this work is a rather singular lipophilic Dox prodrug, which has led to the formation of small-sized nanoassemblies, via the use of the solvent displacement technique and the squalenoylation approach, a strategy that has led to several active nanoassemblies being prepared and a variety of different agents being co-precipitated (theranostic approach) [30,31]. These are soft structures that can be sensitive to a wide range of parameters, meaning that, in order to evaluate the in vitro and in vivo stability improvements in a squalenoyl derivative, it was necessary to efficiently entrap it in phospholipid vesicles (liposomes) [32]. This approach, which combines the intrinsic properties and stability of SLNs with the potency of SQ-Dox, will, of course, be interesting for many, even if significant differences in drug loading must be taken into account for the following in vivo tests. 

The hydrophobic core of SLNs can easily accommodate the Dox prodrug. The preparation procedure, which operates at a temperature that is kept slightly higher than the Krafft point of the chosen BA for a few seconds, does not degrade the prodrug structure and allows significant drug loading to occur. The incorporation of SQ-Dox into the SLN core has been proven by DSC, while size and structure have been confirmed by DLS and AFM analysis. Stability has been confirmed in water for all formulations (SLNs and SQ-Dox NAs). However, NAs showed significant aggregation under conditions resembling cell culture tests.

Activity on resistant ovarian cancer cells was higher for SQ-Dox than it was for Dox, which is similar to results published by Maksimenko et al., who reported in vitro and in vivo activity [8]. However, an experiment on the same cell line, but which possessed induced resistance to Dox, highlights the role played by SLNs in more efficiently delivering SQ-Dox to resistant cells (clone). Hallmarks of apoptosis induction were performed in order to evaluate the apoptotic effect, caspase-3 activation and annexin-V levels of the treatments [33]. Cells that had undergone early apoptosis were precisely tagged in the annexin-V test, which confirmed the earlier results of SQ-Dox’s increased efficiency compared to the free drug, both in normal and resistant A2780 lines [8].

SQ-Dox appeared to be more active than other hydrophobic Dox prodrugs [21], but its improved activity and physical stability against resistant cell lines were more evident when it was incorporated into the SLN core. Moreover, association with lipidic carriers allows SQ-Dox to enter cells even with a low level of LDL receptor expression, as in the case of A2780 Dox-resistant cells [34], and to display significant anticancer efficacy. Indeed, squalenoyl derivative NAs have recently shown that they are preferentially uptaken by cancer cells with a high LDL-accumulating character, which results in higher activity [9,10].

The observation that the inhibition detected in the clonogenic assay was substantially higher than that detected in the cytotoxicity assay suggests that cells, which were still viable after 72 h of treatment, were severely damaged and unable to proliferate in the clonogenic assay, which partially confirms the slow release of the Dox ester from SLNs. Our cell culture viability tests and apoptosis assays show that SQ-Dox NAs displayed antiproliferative and cytotoxic effects that are comparable to those of native Dox. This was proven with the high activity of apoptotic mediators, such as caspase-3 and, even more clearly, with annexin-V.

When all the results presented herein are taken as a whole, it appears clear that SQ-Dox SLNs can partially inhibit the proliferation of a resistant cell line by overcoming MDR, thanks to the fact that the entrapped drug is protected from the Pgp efflux mechanism of a cell. Nevertheless, further investigation into other resistant cancer cell lines, using a variety of LDL receptor expressions, is needed to confirm this hypothesis.

## Figures and Tables

**Table 1:** Physicochemical characteristics of squalenoyl derivative of doxorubicin (SQ-Dox) nanoassemblies (NAs) and of empty and SQ-Dox-loaded solid lipid nanoparticles (SLNs). The stability was assessed via the measurement of particle size after long storage times in water at 4 °C and in complete cell medium (72 h).

Samples	Mean Diameter (nm) ± S.E.	PDI	Zeta Potential (mV) ± S.E.	EE% ± S.E.	Mean Diameter (nm) ± S.E.		
					*T* = 15 *d*	*T* = 45 *d*	*T* = 72 h Cell Medium
SQ-Dox NAs	130 ± 17	0.086	12.12 ± 1.52	-	150 ± 7	160 ± 18	450 ± 35
Empty SLNs	272 ± 6	0.108	−2.20 ± 0.84	-	280 ± 9	282 ± 12	270 ± 12
SQ-Dox SLN1	400 ± 10	0.227	10.34 ± 2.47	85 ± 3	420 ± 5	394 ± 6	400 ± 7
SQ-Dox SLN2	305 ± 6	0.200	2.57 ± 0.43	50 ± 3	348 ± 5	355 ± 5	320 ± 8

**Table 2:** Thermodynamic data determined from the DSC of the NAs and SLNs.

	T_onset_ (°C)	T_m_ (°C)	ΔH (KJ/g)
SQ-Dox NAs	148.09	153.47	141.460
SQ-Dox SLN1	139.28	141.18	49.960
